# Advances in MRGPRX2-mediated anaphylactoid reactions to traditional Chinese medicine injections

**DOI:** 10.3389/fphar.2025.1670739

**Published:** 2025-09-29

**Authors:** Zijin Zhang, Cai Zhang, Runting Yin, Zhen Ouyang, Yuan Gu, Yuan Wei

**Affiliations:** ^1^ School of Pharmacy, Jiangsu University, Zhenjiang, China; ^2^ Chemical Drug Inspection Section, Zhenjiang Center for Food and Drug Inspection and Testing, Zhenjiang, Jiangsu, China

**Keywords:** MRGPRX2, anaphylactoid reactions, traditional Chinese medicine injections, humanized mouse models, genetic polymorphisms

## Abstract

Traditional Chinese medicine injections (TCMIs) play an irreplaceable role in emergency treatment because of their rapid onset and high bioavailability. However, the incidence of non-IgE-mediated anaphylactoid reactions induced by TCMIs is high, accounting for the majority of acute allergic reactions, and posing a serious threat to clinical drug safety. Previous studies have identified the human Mas-related G protein-coupled receptor X2 (MRGPRX2) as the key receptor that mediates these reactions. This review discusses the crucial role of the mast cell surface receptor MRGPRX2 in TCMI-induced anaphylactoid reactions. Regarding research methodologies, approaches utilizing CRISPR/Cas9 technology or hematopoietic stem cell transplantation to construct humanized MRGPRX2 mouse models have been summarized. These models effectively addressed the issue of false negatives caused by species variation. Furthermore, an *in vitro* screening system based on LAD2 cells and the HEK293 overexpression system is described. Combined with calcium influx assays and histamine release measurements, this system enables precise identification of sensitizing bioactive compounds. Clinical studies indicate that MRGPRX2 polymorphisms and racial differences can affect receptor function, potentially altering sensitivity to TCMI-induced anaphylactoid reactions. Optimization strategies have been proposed based on underlying mechanisms, including the implementation of risk-stratified precision medication regimens guided by MRGPRX2 genetic screening. In summary, elucidating MRGPRX2 mechanisms, constructing relevant models, and developing intervention strategies provides a solid scientific foundation for enhancing TCMI safety, offering insights into reducing the risk of clinical anaphylactoid reactions.

## Graphical Abstract

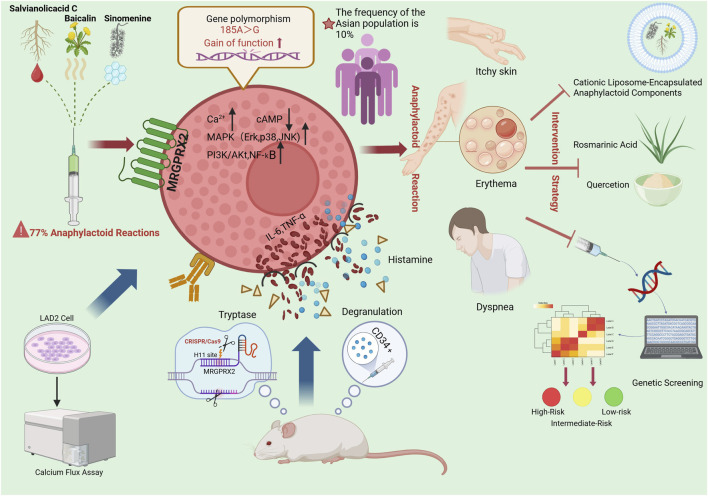

## 1 Introduction

With rapid advancements in traditional Chinese medicine (TCM), TCM injections (TCMIs) have garnered significant attention owing to their definitive therapeutic effects, ability to bypass first-pass metabolism, high bioavailability, and rapid onset of action. They also address the gap in the use of emergency medications in clinical practice (Tu et al., 2021). Nevertheless, the incidence of adverse reactions such as anaphylactoid reactions is influenced by multiple factors, including the complex composition of TCMIs (e.g., active bioactive compounds and excipients), formulation processes (e.g., pH adjustment and solvent selection), dosing regimens (e.g., concentration and infusion rate), and patient-specific physiological conditions (e.g., genetic susceptibility and comorbidities). Collectively, these factors contribute to clinical safety concerns. Anaphylactoid reactions are non-immune-mediated adverse drug reactions that clinically resemble type I allergic reactions but are neither IgE-mediated nor require prior sensitization. They potentially occur upon first exposure (Xu and Dou, 2015). Patients frequently present with symptoms including erythema and edema of the skin and mucous membranes, abdominal pain and diarrhea, dizziness, nausea, and respiratory difficulties. In severe cases, the patients may experience shock, or even death (Kang et al., 2015). However, the exact mechanisms underlying this phenomenon remain unclear. It is potentially associated with drugs that induce the release of bioactive substances, such as histamine, as well as factors such as individual patient variability, drug characteristics, and route of administration. In reports of adverse drug reactions/events (ADR/ADE) related to traditional Chinese medicine (TCM), traditional Chinese medicine injections (TCMI) and oral preparations accounted for 49.3% (with intravenous injections constituting 48.7% of this) and 43.6% of the cases, respectively. Most reactions are anaphylactoid, accounting for approximately 77% of acute allergic reactions (Liang et al., 2015; Liang and Yi, 2020; Li et al., 2019).

Although the mechanism of anaphylactoid reactions caused by TCMIs has not yet been fully elucidated, existing research indicates that human Mas-related G protein-coupled receptor X2 (MRGPRX2) plays a key role in anaphylactoid reactions. MRGPRX2 is a G protein-coupled receptor (that) predominantly expressed on the surface of mast cells. It can be activated by specific components of TCMIs, thereby triggering mast cell degranulation via the G-protein signaling pathway. This process results in the release of inflammatory mediators, including histamine and tryptase, which may induce allergy-like reactions, such as skin erythema, itching, and respiratory difficulties (Kumar et al., 2023). However, because of the differences between the MRGPRX2 homologous gene in rodents and humans, it is difficult to accurately simulate human-like allergic reactions in traditional animal models. This means that the construction of humanized mouse models has become an important research tool (Roy et al., 2021). By replacing the mouse *MrgprB2* gene with the human *MRGPRX2* gene using CRISPR/Cas9 and other gene-editing techniques, a humanized mouse model can be constructed to accurately mimic the pathophysiological processes of human-like allergic reactions and provide a new tool for evaluating the safety of TCMIs.

This review has focused on the key role of MRGPRX2 in mediating anaphylactoid reactions induced by Chinese medicinal injections. Studies have confirmed that several active bioactive compounds (polyphenols, alkaloids) in botanical drug injections can activate MRGPRX2 (Wang J et al., 2024; Che et al., 2022). To precisely resolve this role, the construction of a humanized mouse model of MRGPRX2 is required, and a functional model of mast cells with human receptor expression characteristics has been constructed by replacing the gene using CRISPR/Cas9 technology (Ma et al., 2014), which overcomes the limitation of species-specific differences in the study of non-IgE-mediated anaphylactoid reactions (Bawazir et al., 2025). Using multilevel research, such as *in vitro* cell experiments, *in vivo* animal experiments, and clinical experiments on gene polymorphisms, the receptor affinity and titer intensity of the sensitizing bioactive compounds in TCMIs were revealed. Furthermore, based on the achievements at these different research levels, this study provides a scientific basis for optimizing existing TCMIs and a theoretical basis for precise clinical medication.

## 2 MRGPRX2 structure and function

### 2.1 Receptor structure and gene characterization

MAS-related G protein-coupled receptors (MRGPRs) belong to the class A rhodopsin-like GPCR family. They are primarily expressed in the sensory neurons and innate immune cells, which are typically localized near barrier tissues (Mittal et al., 2019). In humans, the MRGPR family comprises eight distinct receptors, designated as MRGPRX1–MRGPRX4 and MRGPRD–MRGPRG. These receptors detect noxious stimuli and play critical roles in mediating physiological processes, including pruritus (itch), nociception (pain), and innate immune responses (Gour and Dong, 2024).

MRGPRX2, which is located on human chromosome 11p15.1, belongs to the GPCR family and contains 330 amino acids, seven transmembrane helices, and a ligand-binding pocket at the extracellular N-terminus with the key residues Asp-184, Arg-201, and Tyr-279. From the perspective of tissue distribution, MRGPRX2 is highly expressed in the mast cells of the skin, respiratory tract, and intestinal mucosa. This enriched expression pattern makes it a key driver of IgE-independent mast cell activation and degranulation, which, in turn, plays a central role in the regulation of allergic responses and inflammation. However, it is distributed at low levels in sensory neurons and keratinocytes of the central nervous system, and this differential distribution suggesting functional differentiation in different physiological microenvironments (Wollam et al., 2024). When examined from a species-specific perspective, only humans and primates express functional MRGPRX2, a specificity that highlights its unique position in the physiological regulation of higher primates, whereas the rodent homologue MrgprB2 shows significant functional differences (Mcneil et al., 2015).

### 2.2 Physiological and pathological functions

MRGPRX2 is a promising G protein-coupled receptor The ligand-binding site contains multiple structurally conserved hydrophilic amino acid residues and a buried glutamate residue, Glu164, which specifically binds to a wide range of cationic ligands. These ligands include endogenous peptides (neuropeptides and antimicrobial peptides) and various exogenous drugs (fluoroquinolone antibiotics, opioids, phenothiazines, and vancomycin) (Kumar et al., 2021). When these ligands bind to MRGPRX2, the receptor initiates the phospholipase Cβ (PLCβ)–inositol triphosphate (IP3)–calcium ion (Ca^2+^) signaling axis via Gαq/11 protein coupling, leading to a sudden rise in intracellular Ca^2+^ concentration in mast cells, which in turn triggers the release of granular contents (such as histamine, tryptase-like, IL-6), triggering allergy-like phenotypes such as vasodilation, neurogenic inflammation, and tissue edema (Subramanian et al., 2016).

In clinical applications, TCMIs can provoke anaphylactoid reactions due to their complex composition, which includes various active bioactive compounds that directly or indirectly activate MRGPRX2 receptors. Polyphenols, flavonoids, and alkaloids are natural products that can activate MRGPRX2 receptors. *Salvia miltiorrhiza* Bunge (a Lamiaceae plant) is a Chinese medicinal injection commonly used in clinical practice (Lin et al., 2018) to treat cirrhosis, angina pectoris, and other heart diseases (Zhu et al., 2013). It contains three phenolic acids derived from *S. miltiorrhiza*, salvianolic acid A (SA), salvianolic acid C (SC), and isosalvianolic acid C (ISC), which are MRGPRX2 receptor agonists (Lin et al., 2018; Lin et al., 2019). Among these, SC interact most strongly with the MRGPRX2 receptor (Lin et al., 2018). *Scutellaria baicalensis* Georgi (a Lamiacease plant), another Chinese medicine commonly used in clinical practice, contains baicalin, which is used to treat inflammatory and infectious conditions (Bajek-Bil et al., 2023).

However, baicalin has also been implicated in the induction of anaphylactoid reactions (Zhang et al., 2017; Tan et al., 2019; Gao et al., 2018), with studies confirming its capacity to elicit MRGPRX2-dependent reactions in murine models. Furthermore, alkaloids such as codeine, morphine, sinomenine (used for rheumatoid arthritis (Xu et al., 2008), tetrandrine, and pethidine are established MRGPRX2 agonists (Lei et al., 2022). Specifically, tetrandrine exhibits potent agonist activity, with half-maximal effective concentrations (EC_50_) of 2.16 µM in LAD2 human mast cells and 1.84 µM in MRGPRX2-transfected HEK293 cells, significantly enhancing intracellular Ca^2+^ influx and driving mast cell degranulation in both models (Liu et al., 2017).

### 2.3 MRGPRX2 vs. IgE receptor (FcεRI)

The two key pathways for mast cell activation are the MRGPRX2 pathway and the FcεRI (IgE) pathway. Table 1 summarizes the core differences between them.

**TABLE 1:** Comparative analysis of the two mast cell activation pathways.

Characterization	MRGPRX2	FcεRI
Activation methods	Direct ligand binding (sensitization-independent)	IgE cross-linking (requires prior sensitization)
Reaction speed	Within seconds to minutes (rapid-onset response)	Within minutes to hours (delayed-onset response)
Clinical association	Anaphylactoid reaction (can occur upon initial drug exposure)	Classic anaphylaxis (IgE-mediated, triggered upon re-exposure)

## 3 Advances in *in vitro* cellular experimental research

*In vitro* cellular experiments play a crucial role in the study of mechanisms underlying drug-like anaphylactoid reactions. By constructing suitable *in vitro* cell models, the direct effects of herbal injections on mast cells and their potential mechanisms of action can be investigated. The HEK293 overexpression system and LAD2 human mast cell line are important experimental tools (Roy et al., 2021; Mcneil et al., 2015). The HEK293 overexpression system cloned and transfected Mrgprb2 and MRGPRX2 into HEK293 cells. The changes in intracellular Ca^2+^ concentration after drug stimulation were clearly observed using calcium imaging, thus verifying the direct activation of Mrgprb2/MRGPRX2 by the drugs. In a study investigating TCM-induced anaphylactoid reactions, a pharmacological evaluation system identified that SA, ISC, and SC from *Salvia miltiorrhiza* injections significantly stimulated calcium influx in mast cells, with SC demonstrating the most potent agonist activity (EC_50_ = 15.70 ± 4.62 µM, mean ± SEM), suggesting strong ligand–receptor binding affinity to MRGPRX2 (Lin et al., 2018). Furthermore, Liu et al. (2017) investigated sinomenine-induced activation of MrgprB2 and MRGPRX2 using an HEK293 overexpression system. Their findings demonstrated that sinomenine increased the intracellular Ca^2+^ concentration in a dose-dependent manner, exhibiting heightened sensitivity in MRGPRX2-overexpressing cells, with an EC_50_ of 2.77 ± 0.44 µM (Liu et al., 2017).

LAD2 cells are laboratory type-2 cells derived from CD34^+^ cells isolated from the bone marrow of patients with aggressive systemic mastocytosis. These cells have characteristics similar to those of normal human mast cells (Lansu et al., 2017). The LAD2 human mast cell line is an optimal model for studying drug-induced mast cell degranulation and release of mediators. Through β-hexosaminidase release assays and histamine detection, it was demonstrated that multiple traditional Chinese herbal compounds induce degranulation of LAD2 cells and release inflammatory mediators such as histamine. SA, ISC, and SC dose-dependently induced β-hexosaminidase and histamine release from LAD2 cells within 1–100 μM, with SC increasing β-hexosaminidase release from 11% to 26% and elevating histamine levels from 19 ng/mL to 45 ng/mL (Lin et al., 2018). In this study, a response was considered positive if the drug induced a statistically significant (p < 0.05) increase in β-hexosaminidase release ≥10% above the baseline or histamine release ≥15 ng/mL above baseline levels. Baicalin (Han et al., 2017) induced intracellular Ca^2+^ mobilization and histamine release in LAD2 cells, but the induced β-hexosaminidase and histamine release were significantly reduced after knocking down MRGPRX2 expression using siRNA, further confirming the critical role of MRGPRX2 in drug-induced anaphylactoid reactions. In the experiments investigating sinomenine (Liu et al., 2017), LAD2 cells were used to detect sinomenine-induced mast cell degranulation and mediator release. The experimental results showed that sinomenine was able to induce the release of inflammatory mediators such as β-hexosaminidase, histamine, and TNF-α from LAD2 cells in a dose-dependent manner, and this induction could be significantly inhibited by siRNA-mediated knockdown of MRGPRX2 expression, which further confirmed the critical role of MRGPRX2 in sinomenine-induced mast cell activation.

Notably, another study (Han et al., 2017) established a sensitive and rapid liquid chromatography-tandem mass spectrometry (LC-MS/MS)-based method to detect histamine release from LAD2 cells and developed a relative release index to systematically evaluate both the potential and intensity of drug-induced anaphylactoid reactions. This study demonstrated that histamine release from LAD2 cells positively correlated with murine local tissue swelling and Evans blue extravasation intensity, while exhibiting an inverse correlation with the EC_50_ of intracellular calcium influx, conclusively validating the efficacy and reliability of LAD2 cells as a predictive model for evaluating drug-induced anaphylactoid reactions (Han et al., 2017).

## 4 Advances in *in vivo* animal experimental research

### 4.1 Conventional animal models

The construction of animal models can provide an experimental platform for a closer study on the pharmacological effects of TCMIs, safety evaluations, and mechanisms of anaphylactoid reactions, along with a more scientific and reliable theoretical basis for the clinical application of TCMIs. Recent studies conducted worldwide using various animal models, including cynomolgus monkeys, beagle dogs, guinea pigs, rats, and mice, have revealed marked interspecies variations in both the pharmacological sensitivity and clinical phenotypic manifestations of drug-induced hypersensitivity. Meiyu et al. established standardized methodologies employing cynomolgus monkeys and beagle dogs for preclinical assessment of TCMI-induced anaphylactoid reactions (Zhang et al., 2010; Yan et al., 2010). Although large animal models such as cynomolgus monkeys and beagle dogs better recapitulate the clinical manifestations of anaphylactoid reactions, their practical implementation is significantly constrained by species scarcity, exorbitant breeding costs, and single-use limitations for first-exposure reactions. To address this issue, Liang et al. innovatively employed vascular permeability assays (Liang et al., 2012a; Liang et al., 2012b), thereby establishing a groundbreaking protocol that enables reliable evaluation of anaphylactoid responses in rodent models (rats/mice) while overcoming previous translational limitations.

China has officially promulgated the Technical Guidelines for Immunotoxicity Studies (Allergic and Photoallergic Reactions) of Traditional Chinese Medicines and Natural Medicinal Products, establishing a robust regulatory framework that ensures the safety and efficacy of medicinal products. The Systemic Active Anaphylaxis (ASA) assay and its assessment criteria outlined in these guidelines are predominantly used to evaluate type I hypersensitivity reactions; however, their applicability to anaphylactoid reactions (non-IgE-mediated anaphylactoid reactions) remains unaddressed. Subsequent studies have reported that, when evaluating hypersensitivity responses induced by salvianolic acid injections using the ASA model, the observed reactions occurred independently of IgE mediation, thereby providing evidence suggesting the applicability of the ASA model for assessing anaphylactoid reactions (Zhang et al., 2020).

### 4.2 *MRGPRX2*/*Mrgprb2* genes

With the accumulating evidence establishing the pivotal role of the *MRGPRX2*/*Mrgprb2* genes in anaphylactoid reactions, genetically engineered animal models are increasingly being integrated into mechanistic investigations. Despite both being typical seven-transmembrane GPCRs, significant structural divergence exists within their ligand-binding domains, thus leading to critical functional differences. Table 2 compares the structural and functional differences between human MRGPRX2 and murine MRGPRB2 receptors. Previous evidence has consistently indicated that human MRGPRX2 exhibits substantially higher ligand-binding sensitivity than murine MRGPRB2 for most tested pharmacological agents, as demonstrated by differential calcium mobilization profiles and β-hexosaminidase release assays (Mcneil et al., 2015). This species disparity manifests clinically in murine models, where MRGPRB2-dependent responses show marked attenuation or false-negative outcomes in TCMI-induced anaphylactoid reactions, such as cutaneous erythema and vascular leakage. These inherent interspecies variations in receptor sensitivity fundamentally limit the translational relevance of standard murine models for studying human TCMI pathophysiology. Consequently, developing MRGPRX2-humanized murine models has become imperative to accurately recapitulate human anaphylactoid mechanisms.

**TABLE 2:** Comparison of differences between human MRGPRX2 and murine MRGPRB2.

Parameter compared	MRGPRX2 (humans)	MRGPRB2 (mice)
Structural characteristic	Seven transmembrane GPCRs, ligand-binding domain containing a broad-spectrum basic binding pocket Evolutionary history ∼100 million years, binds to human-specific ligands	Seven transmembrane GPCRs with structurally conserved ligand-binding domains Evolutionarily adapted mouse-specific peptide ligands (e.g., beta-defensin)
Ligand-binding specificity	Prefers basic charged molecules (e.g., morphine, vancomycin, chlordiazepoxide)	Primarily recognizes peptide toxins (e.g., mMCP-1)
Drug sensitivity	Low EC_50_ for 80% of the tested drugs (10–100-fold lower than murine-derived receptors) Ligustrazine activation concentration EC_50_ ≈ 10 µM	Low sensitivity to most human drugs (e.g., Ligustrazine EC_50_ > 100 µM) Significant response only to mouse-specific peptides
Signaling activation efficiency	G protein coupling demonstrates high efficiency with potent β-arrestin recruitment capability. Qingkailing injection exhibits an ED_50_ of 0.3 mL/kg in cutaneous reaction models	G protein coupling exhibits low efficiency with a high signaling threshold. Qingkailing injection requires a dose of 5 mL/kg to induce mild vascular leakage
Anaphylactoid reaction model manifestations	Accurately mimics human mast cell degranulation and vascular leakage Highly consistent with clinical drug allergic reactions	False-negative/weak response often seen with herbal injections Phenotypes such as skin erythema and leakage have low relevance to humans

### 4.3 Strategies for constructing humanized mouse models

Current technical approaches for constructing humanized MRGPRX2 receptor mouse models include gene editing-based direct expression and human hematopoietic stem cell transplantation models. Lv (2021) successfully constructed a knockout mouse model of *Mrgprb2* (the murine homolog of MRGPRX2) and discovered that knockout of this gene significantly alleviated anaphylactoid reactions induced by *Houttuynia cordata* Thumb (a Saururaceae plant) injection. CRISPR/Cas9, a molecular biological technology, has been widely used by domestic and foreign scientists for gene editing in various cells and animals owing to its simplicity and applicability (Mcneil et al., 2015). Sun (2023) employed this technique to insert the human *MRGPRX2* gene into the H11 safe harbor locus on mouse chromosome 11, with expression driven by the strong CAG promoter; translation efficiency was further enhanced through Kozak sequence optimization. Subsequently, sgRNA targeting the H11 locus was designed and a donor vector containing the human MRGPRX2 CDS was constructed. Genome editing was performed by pronuclear microinjection into fertilized eggs followed by embryo transfer to pseudopregnant surrogate dams, ultimately yielding F0 founder mice. This technique ensures the consistency and heritability of the gene insertion site, thus guaranteeing a high degree of consistency in the experimental data and avoiding differential expression due to random integration in traditional transgenic technology (Xv et al., 2023). Broad-spectrum promoters such as CAG drive human gene expression in non-target cells such as neurons and epithelial cells, whereas MRGPRX2 functions mainly in human mast cells, which may lead to off-target effects.

Mencarelli et al. successfully established an MRGPRX2-humanized mouse model (Mencarelli et al., 2020). Immunodeficient NOD-scid IL2R*γ*−/− (NSG) mice were selected to transiently express these cytokines through the transplantation of human CD34^+^ hematopoietic stem cells and the introduction of human *GM-CSF* and *IL-3* genes into the mice using the fluid dynamic injection technique to promote proliferation and differentiation of human mast cells. Consequently, the model is suitable for studying phenotypes such as skin erythema and edema triggered by topical injections of drugs or contact allergens. Moreover, the interaction of human-derived mast cells with mouse tissue stromal cells (e.g., endothelial cells, nerve endings) can partially mimic neuro-immune regulation (e.g., itch signaling) in humans. As the results showed, the model successfully generated human mast cells with high MRGPRX2 expression within the mouse skin and other tissues, and its ability to mimic adverse drug reactions was verified by *in vitro* and *ex vivo* experiments, thus providing a new tool for studying non-immune drug allergic reactions. Utilizing this model, MRGPRX2 expression is driven by endogenous regulatory mechanisms, with expression levels, post-translational modifications, and signaling pathway activation patterns more closely aligned with human physiological conditions. However, the model exhibits donor cell heterogeneity and model stability concerns that require further optimization.

## 5 Deciphering mechanisms: *in vitro* and *in vivo* anaphylactoid reaction to TCMIs

### 5.1 Identification of sensitizing components

Chromatographic techniques such as high-performance liquid chromatography [HPLC] and gas chromatography–mass spectrometry have been employed to isolate monomeric bioactive compounds (including alkaloids, saponins, and polysaccharides) and diverse polarity-based fractions from TCMIs. Using *in vitro* cellular models and *in vivo* animal models, the key bioactive compounds of TCMIs that activate MRGPRX2 were identified, and the bioactive molecules responsible for triggering mast cell activation via MRGPRX2 were elucidated, thereby inducing anaphylactoid reactions. In an experimental screening for anaphylactoid bioactive compounds in *Salvia miltiorrhiza* Bunge (a Lamiaceae plant) injections, a novel approach combining a compound mast cell (CMC) line with high MRGPRX2 expression and high-performance liquid chromatography-tandem mass spectrometry (HPLC-MS/MS) was implemented for the first time (Wang et al., 2010), achieving efficient screening and identification of anaphylactoid bioactive compounds (Lin et al., 2018).

### 5.2 Regulation of signal transduction pathways

**FIGURE 1:**
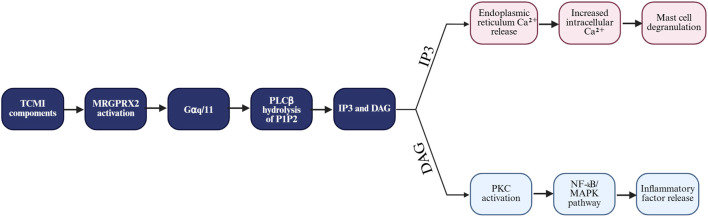
Mechanism diagram.

Anaphylactoid reactions triggered by Chinese medicine injections are mainly mediated by MRGPRX2 receptors, and the mechanism involves the synergistic regulation of multiple signaling pathways (As shown in the mechanism diagram of Figure 1) (Kolkhir et al., 2023). Upon MRGPRX2 receptor activation, phospholipase Cβ (PLCβ) is activated via the Gαq subunit, which catalyzes phosphatidylinositol 4,5-bisphosphate (PIP_2_) hydrolysis to produce inositol 1,4,5-trisphosphate (IP_3_) and diacylglycerol (DAG). IP3 triggers the release of calcium stores by binding to the endoplasmic reticulum IP_3_ receptor (IP3R), creating the “first wave” of calcium transients, while DAG activates protein kinase C (PKC, especially PKCδ-isoforms), which collectively drive a significant increase in intracellular calcium concentrations (Kolkhir et al., 2023). Elevated calcium concentrations contribute to the total degranulation of mast cells and the release of preexisting mediators such as histamine and trypsin-like enzymes through calmodulin-dependent reorganization of the actin backbone and SNARE protein-mediated membrane fusion mechanisms (Metz, 2025).

In addition, MRGPRX2 activation activates the MAPK pathway through the Ras/Raf/MEK cascade, in which the ERK/p38 pathway regulates acute degranulation, whereas the JNK pathway promotes the expression of inflammatory factors, such as IL-6 and TNF-α, by activating AP-1 transcription factor (Ogasawara and Noguchi, 2021). Meanwhile, PKCδ-mediated phosphorylation of IκBα leads to nuclear translocation of NF-κB (p65/p50), which further upregulates the transcription of genes such as TNF-α, IL-8, and others (Quan et al., 2021). Notably, certain TCMI components engage in signaling crosstalk. For instance, the alkaloid sinomenine has been shown to act as an agonist at both MRGPRX2 and Toll-like receptor 4 (TLR4), thus potentially leading to synergistic activation of downstream effectors like NF-κB and amplified inflammatory responses, although the precise molecular interplay warrants further investigation (Wang X et al., 2024; Huang et al., 2022). This multilayered signaling network integrates calcium signaling, MAPK, and NF-κB pathways, not only elucidating the molecular basis of acute anaphylactoid reactions induced by TCMIs but also providing mechanistic insights into chronic inflammatory processes such as monocyte infiltration and tissue fibrosis. Furthermore, it establishes a theoretical framework for targeted interventions, including the development of MRGPRX2 antagonists and PKCδ inhibitors.

### 5.3 Mechanisms of mast cell activation

As detailed in Section 2.2, the TCMIs containing specific herbal bioactive compounds robustly activate mast cell-surface MRGPRX2 receptors, triggering the Gαq-PLCβ signaling pathway and leading to rapid PIP_2_ hydrolysis into IP_3_ and diacylglycerol (DAG) (Kolkhir et al., 2023). This initiates Ca^2+^ release from endoplasmic reticulum stores, followed by sustained Ca^2+^ influx mediated by STIM1/Orai1 channels, culminating in the rapid elevation of intracellular Ca^2+^ concentrations exceeding 500 nM (Metz, 2025). Building on this Ca^2+^ signal, activated mast cells undergo degranulation via distinct pathways.

DAG activates protein kinase C (PKC). PKC synergizes with calmodulin to induce actin cytoskeleton reorganization and generates RhoA/ROCK-mediated contractile forces. This process orchestrates Rab3D-regulated translocation of preformed granules to the plasma membrane. The subsequent fusion of granules with the plasma membrane is mediated by SNARE complexes (e.g., SNAP23/Syntaxin4) (Kolkhir et al., 2023). This cascade results in the explosive exocytosis of mediators (e.g., histamine and tryptase) within 5–15 min, precipitating acute anaphylactoid reactions characterized by vasodilation and bronchoconstriction. Pharmacologically, this acute response can be inhibited by Ca^2+^ chelators (e.g., BAPTA-AM) or SNARE disruptors (e.g., tetanus toxin), suggesting potential strategies for enhancing TCMI safety.

Under prolonged low-dose TCMI stimulation, mast cells may undergo piecemeal degranulation. This process primarily involves vesicular transport regulated by Rab GTPases (e.g., Rab5, Rab7), facilitating selective mediator shuttling via LAMP1^+^ vesicles (Hutagalung and Novick, 2011; Martinez-Arroyo et al., 2021). Unlike acute degranulation, this mode manifests as a sustained, selective release of mediators, including the pruritogenic cytokine IL-31, pro-angiogenic factor VEGF, and monocyte chemoattractant CCL2/MCP-1. This sustained release pattern may contribute to long-term pathological sequelae such as chronic pruritus, neovascularization, and tissue mononuclear cell infiltration. Ultrastructural hallmarks of piecemeal degranulation, observable by transmission electron microscopy, include characteristic granule “vacuolation.” Single-cell RNA sequencing can further delineate the differential secretory profiles of specific mast cell subpopulations involved in this process. This mechanism provides a molecular basis for chronic adverse effects associated with long-term TCMI administration.

Utilizing *in vivo* animal models combined with methodologies like Ca^2+^ flux assays enables the precise identification of MRGPRX2-activating components within TCMIs. This approach also facilitates the dissection of the molecular mechanisms underpinning MRGPRX2-mediated anaphylactoid reactions, encompassing Ca^2+^ mobilization via the Gαq-PLCβ pathway, PKC activation, and inflammatory mediator expression regulated by MAPK and NF-κB signaling. In addition, it is possible to study mast cell activation mechanisms, such as acute degranulation, which leads to acute anaphylactoid reactions and chronic pathological changes triggered by progressive release.

## 6 Clinical advances: genetic polymorphisms and anaphylactoid risk

### 6.1 Functional impact of *MRGPRX2* polymorphisms on anaphylactoid reactions

#### 6.1.1 Gain-of-function mutations potentiate receptor activity

Research by Professor Geng Songmei at Xi’an Jiaotong University revealed that in patients with chronic spontaneous urticaria, the 185A > G single-nucleotide mutation in MRGPRX2 significantly enhanced the receptor function (Ye et al., 2025). *In vitro* experiments demonstrated that mast cells transfected with the 185A > G mutant gene exhibited a 30% increase in histamine release, significantly elevated β-hexosaminidase activity, and a 50% upregulation in receptor expression. The mutation promoted the phosphorylation of the PLC-γ/IP3R and PI3K/AKT pathways, suggesting that it amplifies signaling through enhanced G protein coupling efficiency. Patients with chronic spontaneous urticaria harboring this mutation have significantly higher disease activity scores (UAS7) and lower peripheral blood basophil and lymphocyte counts, suggesting that the mutation may exacerbate the disease through immune regulatory imbalance (Cuc et al., 2025).

The research team also identified a compound mutation (185A > G + 46A > C) with a detection rate of 28.75%. However, this variant did not exhibit the same functional enhancement effect as a single mutation (185A > G). The 46A > C mutation resides in a non-coding region or non-critical structural domain, likely preventing the synergistic enhancement of receptor activity. In contrast, the 185A > G mutation is located within the coding region and directly affects coupling efficiency between the transmembrane domain of the receptor and G proteins. The high detection rate of co-mutations suggests other undiscovered regulatory functions in this population, such as indirect effects on MRGPRX2 expression via epigenetic or post-transcriptional modifications.

Further extrapolation suggests that patients harboring the 185A > G mutation may exhibit a heightened risk of anaphylactoid reactions to TCMIs. The combination of such mutations, which significantly increases patient sensitivity to histamine, and herbal injections, which are high-risk factors for anaphylactoid reactions, significantly increases the risk of serious adverse reactions. When patients carrying the 185A > G mutation are treated clinically with herbal injections, even relatively low concentrations of the injection components that cause histamine release may trigger a dramatic reaction in patients with the mutation receptor. Symptoms can develop rapidly and severely. Therefore, it is strongly recommended that tests for 185A > G and other related genes be performed before administering herbal injections to patients with a history of severe allergies and adverse drug reactions (especially reactions to multiple drugs or herbal injections), to clarify whether the patients are carriers. This study provides a new approach for the clinical application of TCMIs to reduce the risk of class allergies.

#### 6.1.2 Interactions between TCMI components and MRGPRX2 SNPs

Single-nucleotide polymorphisms (SNPs) are the most common form of genetic polymorphism, and refer to variants of individual nucleotides in genomic DNA sequences that are widespread and highly stable in the genome (Zhao et al., 2014). SNPs in MRGPRX2 may lead to changes in the amino acid sequence encoded by the gene, thereby affecting the structure and function of the receptor (Yang et al., 2005). Relevant studies indicate that RBL-2H3 cells expressing any of four missense SNPs (Gly165Glu, Asp184His, Trp243Arg, His259Tyr) in MRGPRX2 exhibit abolished responsiveness to ligands—including substance P, hemokinin-1, human β-defensin-3, and icatibant. Patients carrying these SNPs may be protected from drug-induced mast cell degranulation and hypersensitivity reactions (Porebski et al., 2018). Molecular docking simulations suggest that the G165E mutation may disrupt charge distribution within the ligand-binding pocket, thereby weakening hydrogen bonding or hydrophobic interactions (Ratnayake et al., 2024). However, further investigation is warranted to determine how these SNPs alter the ligand-binding properties of MRGPRX2 (Alkanfari et al., 2018). Such mutations may reduce the risk of anaphylactoid reactions to herbal injections and provide new ideas for circumventing these reactions during the use of herbal injections.

Some bioactive compounds present in botanical drug injections, such as peptides and sesquiterpenoids, trigger mast cell degranulation by directly binding to the ligand-binding domain of the MRGPRX2 receptor via specific charge distribution or hydrophobic moieties (Roy et al., 2021). Germacrone, a sesquiterpenoid compound extracted from *Curcuma wenyujin* Y.H.Chen et C.Ling (a *Zingiberaceae* plant) (Gao et al., 2019), binds directly to the MRGPRX2 receptor in mast cells. Its specific charge distribution and hydrophobic moieties enable interactions with the ligand-binding domain of the receptor. Experimental studies have confirmed that germacrone induces a dose-dependent increase in the intracellular calcium concentration in mast cells. It activates LAD2 cells and triggers the release of degranulation markers, including β-hexosaminidase and histamine (Gao et al., 2019). In clinical practice, patients carrying protective SNP variants, such as G165E, exhibit a significantly reduced risk of anaphylactoid reactions when administered TCMIs containing peptides or sesquiterpenoid compounds (e.g., germacrone). This supports the implementation of pharmacogenomic-guided precision administration.

Gene polymorphisms may also affect the interaction of MRGPRX2 with G proteins or other signaling molecules, which in turn may alter the degree and rate of activation of signal transduction pathways. This study indicated that V282M (Val282Met) and V123F (Val123Phe) mutations localize to the transmembrane domains or intracellular loops of the receptor, which are critical for G protein coupling (Chompunud et al., 2019). These variants impair the coupling efficiency between MRGPRX2 and G proteins (e.g., Gαq/11). The V282M mutation may enhance the receptor–G protein coupling efficiency, thus facilitating rapid downstream signal transduction. In contrast, the R138C (Arg138Cys) and R141C (Arg141Cys) mutations likely disrupt the electrostatic interactions between the receptor and G proteins, thereby impairing coupling efficiency and resulting in delayed or diminished signal transduction (Chompunud et al., 2019). Signal transduction-enhancing mutations may amplify the activating effects of herbal components on mast cells. Clinically, patients harboring the V282M mutation exhibit exacerbated anaphylactoid reactions to highly sensitizing TCMIs such as *Houttuynia cordata* Thumb (a Saururaceae plant) and Shuanghuanglian injections. The clinical manifestations included an expanded erythema area (+35%) and a prolonged duration of edema (+50%). In contrast, patients with the V123F mutation may exhibit enhanced tolerance to low doses of the drug. However, prolonged drug use may increase the risk of receptor desensitization.

### 6.2 Ethnic variation analysis of MRGPRX2 mutation spectra

The 185A > G single-nucleotide variant of MRGPRX2 has a detection frequency of 10% in Asian populations. This mutation significantly enhances receptor activity, leading to elevated mast cell degranulation capacity and increased phosphorylation levels in downstream signaling pathways (Ye et al., 2025). Furthermore, the V282M (Val282Met) mutation was localized in transmembrane domain 6 (TM6). By enhancing hydrophobic interactions with Gαq/11, it increases G protein-coupling efficiency, thereby amplifying the anaphylactoid effects induced by TCMIs (Wang et al., 2022). Songmei et al. identified a compound mutation (185A > G + 46A > C) with a population frequency of 28.75% in an Asian population. Notably, this variant did not enhance receptor function, akin to the 185A > G single mutation, indicating the position-dependent specificity of the mutational effects on receptor activity. This functional divergence may be associated with distinct gene–gene interactions or epigenetic regulatory mechanisms inherent in Asian populations (Cuc et al., 2025).

Relatively few studies have investigated MRGPRX2 mutation profiles in Caucasian populations. Most available studies have focused on validating the receptor function, such as mouse homozygous knockout experiments, to confirm its central role in anaphylactoid reactions. Targeted investigations of the functional MRGPRX2 variants prevalent in Caucasian populations, such as R138C (Arg138Cys) and R141C (Arg141Cys), remain scarce. Several reports have suggested that these mutations may disrupt receptor–G protein electrostatic interactions, resulting in delayed signal transduction (Mcneil et al., 2015). The All-of-the-Us Research Program revealed that the genetic ancestry of the US population is predominantly European (66.4%), with Asian ancestry accounting for 7.6%. This Eurocentric bias in genomic studies has resulted in the underrepresentation of non-European demographic groups (e.g., Asians) in MRGPRX2 mutation databases, potentially masking cross-population functional variations (Sharma et al., 2025). For instance, the *NFE2L2* gene mutation, which is associated with oxidative stress response and is highly prevalent in Asian populations, is rarely observed in Caucasian cohorts. This suggests that analogous mechanisms underlie the differential mutational spectra of MRGPRX2 across populations (Mukherjee et al., 2025). Table 3 summarizes corresponding genetic polymorphisms across ethnic groups.

**TABLE 3:** Genetic polymorphism disparities between asians and caucasians.

Parameter compared	Asian populations	Caucasian populations	Summary
Main mutation types	Predominantly gain-of-function mutations (e.g., 185A > G, V282M)	Predominantly loss-of-function mutations (e.g., R138C, R141C)	Mutations in Asian populations predominantly enhance receptor activity, whereas those in Caucasian populations may attenuate signal transduction
Detection rate	185A > G single mutation (10%) 185A > G + 46A > C compound mutation (28.75%)	Insufficient data; detection rates of mutations such as R138C/R141C remain undetermined	The mutational spectrum of MRGPRX2 is relatively well-characterized in Asian populations, whereas data remain scarce for Caucasian cohorts
Functional impact	185A > G: Enhances receptor activity V282M: enhances G protein-coupling efficiency	R138C/R141C: Disruption of receptor–G protein electrostatic interactions (delayed signaling)	Asian variants amplify the anaphylactoid effects of TCMIs, whereas Caucasian mutations may attenuate immediate reactions but increase the risk of tolerance induction to therapeutic desensitization
Clinical association	Significantly higher risk of anaphylactoid reactions	Data on hypersensitivity risks remain insufficient, which may be related to the type of drug and differences in exposure	For Asian populations receiving high-risk anaphylactoid reaction-inducing injections (e.g., *Houttuynia cordata* injection), genotype-based screening should be prioritized. In Caucasian cohorts, dose optimization guided by pharmacogenomic profiling is necessary

## 7 Optimization strategies for existing TCMIs

### 7.1 Safety assessment of TCMIs

By analyzing the relationship between the drug dosage and biological effects, key safety parameters, including EC_50_, Hill coefficient, and maximum effect, were accurately determined (Lei et al., 2022). These parameters provide an important basis for evaluating the potency, efficacy, and safety of drugs. The therapeutic index (LD_50_/EC_50_) was used to further evaluate drug safety. Integrating *in vitro* cellular assays with physiologically based pharmacokinetic modeling enables the simulation of drug absorption, distribution, metabolism, and excretion. This methodology facilitates the quantitative prediction of *in vitro*-to-*in vivo* dose translations for clinical administration (Li et al., 2025; Wang et al., 2025). This combination not only improves the accuracy of drug safety assessment but also provides a more reliable dosage prediction for the clinical application of TCMIs.

The MRGPRX2-humanized mouse model provides a unique platform to elucidate the synergistic mechanisms of compound formulations in TCMIs. By overexpressing human MRGPRX2 in murine mast cells, this system recapitulates human physiological and pathological states, enabling experimental investigations under near-physiological conditions. This model enables the deconvolution of synergistic interactions among multicomponent formulations in TCMIs, revealing how individual constituents either activate or inhibit the MRGPRX2 receptor to modulate mast cell function, thereby regulating immune responses and inflammatory cascades. Clinically, when TCMIs are administered, certain constituents promote mast cell degranulation via MRGPRX2 activation (release of inflammatory mediators), whereas others exert inhibitory effects. This dynamic equilibrium determines the net therapeutic efficacy of the compound formulation.

Traditional *in vivo* experiments using animal models are also important to assess the safety and efficacy of herbal compounds. By monitoring physiological and pathological changes in animal models after administration of TCMIs, we were able to better understand the risk of analogous allergies caused by TCMIs and optimize the composition of compounded formulas to improve the safety of their clinical application. More importantly, this model adheres to internationally recognized GLP specifications (Mcdorman et al., 2024), which provide a standardized assessment framework for the study of TCMIs, help promote the modernization of TCMs, and make it more compliant with the requirements of international drug regulatory agencies, thus facilitating the entry of TCMIs into the international market.

### 7.2 Safety screening and targeted intervention for TCMIs

Targeted research experiments on MRGPRX2 play a bidirectional role in the development of TCMIs: on the one hand, it serves as a screening platform for sensitizing bioactive compounds so that potential risk molecules activating MRGPRX2 are accurately identified, and on the other hand, it provides a functional validation tool for developing targeted antagonists and optimizing the formulation process. The MRGPRX2-humanized mouse model was used to reproduce the pharmacological characteristics of human receptors and completely retain the pharmacological characteristics of the receptors and signaling features, thus simulating the human pathological reactions of the anaphylactoid reaction research platform, which can systematically assess the direct activation effects of TCM bioactive compounds on mast cells. We proposed a three-tiered evaluation system for pharmaceutical screening. The primary screening phase employs *in vitro* high-throughput calcium flux assays utilizing fluorescent calcium indicators to detect dynamic changes in intracellular Ca^2+^ concentrations (Lin et al., 2018), which enables the rapid identification of potential MRGPRX2 agonists in TCMIs. Subsequently, the sensitization intensity of the candidate bioactive compounds was quantitatively assessed *in vivo*, and changes in the skin microcirculation were dynamically monitored and analyzed in conjunction with mouse behavior to quantify allergy-related manifestations. Inflammatory mediator profiles, such as serum histamine, tryptase-like enzyme, and prostaglandin D2 levels, were also precisely determined, and a multidimensional component-receptor-phenotype correlation map was constructed. For instance, in mechanistic studies of Shuanghuanglian injection-induced sensitization, baicalin and forsythoside A were successfully identified as high-affinity ligands for MRGPRX2 through rigorous screening (Chen et al., 2021).

The intervention strategies developed for TCMIs provide key experimental scenarios for targeted drug design and innovation in the formulation process. Virtual screening based on MRGPRX2 crystal structure was combined with *in vivo* pharmacodynamic validation of humanized models. Researchers have used a proprietary chemical library comprising approximately 12,000 compounds to screen for MRGPRX2 modulators. Using a stable MRGPRX2-expressing HEK293 cell system, we conducted substance-induced intracellular calcium mobilization assays. This approach identified two distinct heterocyclic compounds, designated as compounds 1 and 2, as candidate modulators (Ogasawara et al., 2019). Both compounds block intracellular calcium mobilization mediated by basal secretagogues and substance P-induced MRGPRX2 in a concentration-dependent manner, and can also block the GTP-γS-binding activity of Gα proteins downstream of MRGPRX2, suggesting that they are competitive antagonists of the MRGPRX2 receptor (Ogasawara et al., 2019). A research team at the University of Bonn (Germany) has developed PSB-172656, a sub-nanomolar MRGPRX2 receptor antagonist. This compound potently inhibits mast cell degranulation and significantly attenuates inflammatory responses in murine models (Al et al., 2025).

For intelligent delivery system design, we propose utilizing cationic liposome encapsulation technology to modulate the surface charge of sensitizing bioactive compounds (Mainardes and Silva, 2004). This modification prevents their interaction with the positively charged ligand-binding pocket of MRGPRX2 (Grimes et al., 2019). Concurrently, screening for natural bioactive compounds with MRGPRX2 inhibitory activity is recommended. Experimental evidence demonstrates that Rosmarinic acid suppresses MRGPRX2-mediated anaphylactoid responses (Ding et al., 2023). Another promising anti-inflammatory and anti-allergic natural product, fisetin (Hada et al., 2021)—is abundant in fruits and vegetables (Terahara, 2015). It inhibits multiple signaling pathways *in vitro*, including PI3K-Akt-mTOR, p38 MAPK, and NF-κB cascades (Ren et al., 2020; Adhami et al., 2012), all implicated in its suppressive effects observed in human inflammatory skin models. Mechanistic studies indicated that fisetin exerts its inhibitory effect by binding to the orthosteric site of MRGPRX2, thereby blocking mast cell activation (Jia et al., 2022). The co-administration of these inhibitory constituents with sensitizing bioactive compounds leverages receptor steric hindrance effects to mitigate activation risks. These advancements not only provide a scientific foundation for enhancing the safety profile of TCMIs but also pioneer novel pathways for targeted drug design and formulation technology innovation. Collectively, they have propelled the evolution of TCMIs from empirical risk management to precision-targeted modulation.

### 7.3 Precision medication

Previous studies targeting MRGPRX2 polymorphisms have provided insights into the precise administration of medications to patients. For example, patients must undergo genetic screening and risk assessment before medication administration. Screening for gain-of-function mutations prevalent in Asian populations (e.g., 185A > G and V282M) and protective mutations (e.g., G165E and D184H) must be prioritized. Next-generation sequencing and Sanger sequencing should be used to construct personalized genetic profiles for clinical risk stratification. Patients carrying functionally acquired mutations (e.g., 185A > G detection rate of 10%) are labeled as high-risk, and it is recommended that herbal ingredients that may activate MRGPRX2 be avoided and medication restrictions be relaxed for patients carrying protective mutations.

Genetic screening and risk assessment provide critical support for clinical decisions regarding pharmacotherapy. Using cell membrane chromatography to characterize the interaction profiles between TCM bioactive compounds and mutant MRGPRX2 receptors, we established a database of high-risk bioactive compounds such as cationic peptide-containing TCM extracts. Concurrently, real-time dose adjustment was implemented using a graded dose escalation protocol for high-risk patients. Sandwich ELISA kits (e.g., with patented peptide-specific antibodies) were used to quantify MRGPRX2 protein expression in peripheral blood. Clinical interventions must be initiated when the expression levels exceed the baseline values by two-fold. The real-time response of patient-derived mast cells to TCMIs may also be evaluated using calcium flow monitoring or fluorescent probe labeling, allowing us to create an early warning system of “degranulation threshold” for the real-time control of drug administration.

## 8 Discussion

TCMIs have irreplaceable advantages in the treatment of acute illnesses; however, the anaphylactoid reactions caused by them are of increasing concern (Tu et al., 2021). This review systematically summarizes the pivotal role of MRGPRX2 in anaphylactoid reactions induced by TCMIs. By integrating the findings from *in vitro* cellular assays, *in vivo* animal models, and clinical genetic polymorphism studies, this study offers new perspectives on the safety profiles of TCMIs. Based on current research advances, we discuss the related issues below.

### 8.1 Central role of MRGPRX2 in anaphylactoid reactions

The MRGPRX2 receptor, an important GPCR expressed on mast cells (Tatemoto et al., 2006), can be directly activated by TCMI bioactive compounds, such as polyphenols and alkaloids (Liu et al., 2017; Wang et al., 2020; Bhambhani et al., 2021), which triggers an IgE-independent mast cell degranulation response. These findings provide a molecular basis for the high incidence of TCMI-induced anaphylactoid reactions. However, the ligand recognition mechanism of MRGPRX2 is not completely understood. In particular, the dynamic equilibrium mechanism, whereby multiple bioactive compounds within TCM formulations synergistically activate or inhibit receptors, requires further elucidation. Although the high promiscuity of the MRGPRX2 receptor enables it to recognize a diverse array of endogenous and exogenous ligands, the specific structural characteristics of its ligand-binding domain require further elucidation. Future studies employing techniques such as cryoelectron microscopy and molecular dynamics simulations could elucidate the binding modes of MRGPRX2 to TCMI bioactive compounds. This elucidation provides a structural basis for designing specific antagonists.

The potential crosstalk of MRGPRX2 with other immune receptors such as TLR4 and FcεRI (West and Bulfone-Paus, 2022; Elieh et al., 2020) may further amplify inflammatory reactions. Evidence suggests compounds like sinomenine can activate both MRGPRX2 and TLR4 (Wang J et al., 2024; Huang et al., 2022), potentially leading to synergistic NF-Κb activation. However, the molecular mechanisms governing this multireceptor cooperative network, its contribution relative to MRGPRX2 alone in TCMI reactions, and its role in chronic sequelae remain largely unexplored (Kolkhir et al., 2023). Recent reviews (Gour and Dong, 2024; Quan et al., 2021) broadly discuss MRGPRX2’s role in immunity, but often underemphasize this specific crosstalk in the context of TCMI toxicity. This review has aimed to fill this gap. Future studies should prioritize the elucidation of these complex interactions.

### 8.2 Advantages and limitations of current methodological approaches

*In vitro* cellular experiments can directly and specifically study the interactions of herbal components with MRGPRX2 receptors and are characterized by the easy manipulation of gene editing and other operations that can finely resolve signaling pathways. This approach is suitable for the rapid and cost-effective screening of large libraries of TCMIs or their isolated constituents to assess their activation potential on MRGPRX2, enabling preliminary tiered risk assessment. However, *in vitro* cellular assays have certain limitations. They cannot simulate the absorption, distribution, metabolism, and excretion of drugs *in vivo*, and drug prototypes and metabolites may have different activities. Furthermore, these assays typically utilize mast cell lines or primary cells but fail to account for the phenotypic and functional heterogeneity of mast cells derived from different tissues, as well as their interactions with other immune cells. Recent reviews have acknowledged these limitations but seldom delve into their specific impact on TCMI safety prediction accuracy.

MRGPRX2-humanized mouse models, generated via genetic editing or hematopoietic stem cell transplantation (Sun, 2023; Mencarelli et al., 2020), successfully circumvent the issue of false-negative results arising from species differences in conventional animal models. Thus, these models provide an experimental platform that closely approximates human physiological conditions for evaluating the safety of TCMIs. Despite the significant advantages of humanized mouse models in mimicking human anaphylactoid reactions, their applicability in pathological processes such as long-term chronic inflammation and tissue fibrosis still needs to be validated. Future studies should evaluate the role of MRGPRX2 during the long-term use of herbal injections by constructing a chronic inflammation model. Currently, the construction of MRGPRX2-humanized mouse models faces challenges related to expression specificity and functional stability. Recent reviews (Roy et al., 2021; Gour and Dong, 2024) have highlighted the promise of humanized models but often provide a less critical analysis of their current technical bottlenecks and translational readiness compared to this focused discussion on TCMI applications. Future studies could further enhance the physiological relevance of these models by optimizing gene-editing techniques or employing tissue-specific conditional knockout strategies.

### 8.3 Clinical risk-mitigation strategies for anaphylactoid reactions

Building on MRGPRX2 research findings, clinical strategies to mitigate the risks of anaphylactoid reactions associated with TCMIs may include the following approaches: component screening and structural modification, utilization of humanized models for high-throughput screening of anaphylactoid components, and chemical modification to reduce their affinity for MRGPRX2. The development of targeted antagonists offers a novel strategy for optimizing TCMIs. For instance, the discovery of natural antagonists such as rosmarinic acid and fisetin (Mainardes and Silva, 2004) not only provides new directions for designing compound formulations but also prompts researchers to explore their detoxification mechanisms through combinatorial formulations with sensitizing components. This approach aims to maintain the therapeutic efficacy while minimizing adverse reactions. Innovation in delivery systems is an important direction for optimizing TCMIs. The application of smart carriers is expected to realize precise delivery of sensitizing ingredients and reduce nonspecific activation, further improving the safety of TCMIs.

Genetic polymorphisms in MRGPRX2 can significantly alter receptor function, leading to interindividual variability in the response to TCMIs. Integrating patient genetic screening, future research could establish a “genotype-dose-response” association database. This study provides an evidence-based foundation for identifying high-risk individuals and guiding personalized medication strategies in clinical practice. Genetic polymorphisms not only affect the function of the MRGPRX2 receptor, but also interact with the patient’s genetic background and environmental factors to further modulate the occurrence of anaphylactoid reactions. While other reviews have discussed polymorphisms, this review uniquely links them explicitly to TCMI risk stratification and practical hurdles in implementing genetic screening in diverse healthcare settings. Future research should employ integrated multiomics approaches (e.g., genomics, transcriptomics, and metabolomics) to comprehensively dissect the underlying mechanisms by which *MRGPRX2* genetic polymorphisms contribute to anaphylactoid reactions induced by TCMIs. This comprehensive approach will help reveal how genetic polymorphisms affect individual responses to TCMIs in complex environments and provide a solid theoretical basis for precision medicine and individualized medication.

### 8.4 Multidimensional challenges in clinical translation of MRGPRX2 research

#### 8.4.1 Technical hurdles and unresolved scientific questions

Research on MRGPRX2 presents novel pathways for enhancing Traditional Chinese Medicine injection (TCMI); however, clinical translation is confronted with significant technical and scientific hurdles. Humanized models capable of simulating human receptor function have exhibited persistent limitations, including expression heterogeneity (encompassing off-target expression in non-target cells), long-term instability, and prohibitive costs and timelines for large-scale screening. The interaction mechanisms between human mast cells and murine stromal components (such as endothelial cells and nerve endings) remain unclear, potentially compromising the fidelity of the neuroimmunomodulatory pathway. Furthermore, crosstalk between MRGPRX2 and receptors like TLR4 and FcεRI may amplify inflammatory responses; however, the molecular mechanisms governing this multi-receptor regulatory network are poorly characterized. Quantitative studies elucidating how multi-bioactive compound TCMIs dynamically regulate MRGPRX2 through homeostatic mechanisms (synergistic activation/inhibition) are lacking, thereby hindering precise formulation optimization.

Critically, TCMI-induced anaphylactoid reactions constitute a multifactorial process, wherein the MRGPRX2 activation represents only one key mechanism. Formulation additives (e.g., Cremophor EL) independently activate MRGPRX2, whereas alkaline pH ( > 8.5) in certain injections directly induces mast cell histamine release. The clinical severity was dose dependent, as evidenced by the sigmoidal relationship between serum baicalin levels and erythema severity (*R* ^2^ = 0.79, p < 0.001). Patient-specific factors, such as renal impairment, which prolongs sinomenine half-life by 3.2-fold, significantly increase the risk of cumulative anaphylactoid reactions. Therefore, deconvoluting these complex interactions necessitates future research adopting integrated “component-formulation-patient” models.

Furthermore, beyond direct activation of MRGPRX2, certain processing methods for botanical drugs—such as delayed drying or inadequate sterilization—may introduce contaminants like lipopolysaccharides (LPS). LPS activates Toll-like receptor 4 (TLR4) on mast cells and other immune cells, potentially potentiating MRGPRX2-mediated responses and amplifying inflammatory cascades. Future quality control protocols should incorporate LPS quantification to ensure batch-to-batch consistency and product safety, thereby minimizing unintended immune activation.

#### 8.4.2 Cost-effectiveness and clinical Implementation barriers

Precision pharmacotherapy based on MRGPRX2 genetic polymorphisms requires standardized genetic screening. However, current sequencing methodologies exhibit prohibitive costs and protracted turnaround times, which impede their implementation in resource-constrained regions. Preventive screening for high-risk variants (e.g., c.185A > G in Asian cohorts with 10% prevalence) demonstrates efficacy in mitigating anaphylactoid reactions; however, its health economic value remains unquantified owing to insufficient cost-effectiveness analyses (CEA) to inform clinical decision pathways. Substantial developmental bottlenecks still persist in point-of-care testing (POCT) technology: Detection of MRGPRX2 functional mutations (e.g., p.V282M) requires simultaneous multiplexed SNP genotyping. Current POCT platforms (e.g., lateral flow chromatographic strips) fail to achieve high-sensitivity multiplex detection (<1% false-negative rate) within 15-min operational windows. Furthermore, no POCT device has undergone comprehensive clinical validation of the integrated “near-patient testing → therapeutic decision-making → prognostic enhancement” pathway, resulting in deficient real-world performance evidence.

#### 8.4.3 Implementation constraints of racial/ethnic and environmental disparities

Current analyses on the risk of MRGPRX2 mutations exhibit significant population representation gaps. While this study highlights the clinical implications of the Asian-enriched gain-of-function variant 185A > G (10% allele frequency), a more critical reality must be confronted: mutation landscapes remain virtually uncharacterized in African, Latin American, and Indigenous populations. Because of their unique demographic histories, African-ancestry groups may harbor undiscovered MRGPRX2 functional variants, such as high-frequency population-specific mutations in immune-related genes (e.g., IL4R and ADRB2) identified in African cohorts, thus suggesting that analogous mechanisms may exist in MRGPRX2. Neglecting such variants introduces bias in the risk stratification of anaphylactoid reactions.

Moreover, European populations predominantly carry loss-of-function mutations (e.g., R138C/R141C) that may attenuate drug responses, whereas Asian populations exhibit gain-of-function variants that exacerbate the risk of anaphylactoid reaction. Consequently, population-stratified pharmacotherapy is warranted to mitigate adverse outcomes.

Although anaphylactoid reactions are modulated by multifactorial regulators (e.g., epigenetics and the gut microbiome), current research fails to integrate multi-omics data (epigenomic, metabolomic, etc.), precluding the elucidation of gene-environment interactions underlying cross-population disparities.

Integrating multidisciplinary approaches, such as genetic testing, component optimization, real-time monitoring, and the development of novel delivery systems, can significantly reduce the risk of anaphylactoid reactions to TCMIs. Future research should explore the TCM-modulated targets within the MRGPRX2 signaling pathway. Combining this with artificial intelligence-driven predictive modeling will advance the implementation of precision medicine frameworks for TCMI safety. The MRGPRX2-humanized mouse model is a powerful tool for mechanistic studies and safety enhancement of TCMIs. However, its application faces many technical bottlenecks that must be resolved through multidisciplinary collaboration, thus promoting a transition from basic research to clinical practice. Specifically, optimizing the TCMI composition, developing targeted antagonists, and innovative delivery systems, combined with genetic polymorphism analysis and individualized dosing strategies, are expected to provide safer and more effective solutions for the clinical application of TCMIs.
